# A Highly Robust Encoder–Decoder Network with Multi-Scale Feature Enhancement and Attention Gate for the Reduction of Mixed Gaussian and Salt-and-Pepper Noise in Digital Images

**DOI:** 10.3390/jimaging11020051

**Published:** 2025-02-10

**Authors:** Milan Tripathi, Waree Kongprawechnon, Toshiaki Kondo

**Affiliations:** School of Information, Computer and Communication Technology, Sirindhorn International Institute of Technology, Thammasat University, Pathum Thani 12120, Thailand; d6722300024@g.siit.tu.ac.th (M.T.); tkondo@siit.tu.ac.th (T.K.)

**Keywords:** image denoising, Gaussian, salt-and-pepper, encoder–decoder, multi-scale feature enhancement

## Abstract

Image denoising is crucial for correcting distortions caused by environmental factors and technical limitations. We propose a novel and highly robust encoder–decoder network (HREDN) for effectively removing mixed salt-and-pepper and Gaussian noise from digital images. HREDN integrates a multi-scale feature enhancement block in the encoder, allowing the network to capture features at various scales and handle complex noise patterns more effectively. To mitigate information loss during encoding, skip connections transfer essential feature maps from the encoder to the decoder, preserving structural details. However, skip connections can also propagate redundant information. To address this, we incorporate attention gates within the skip connections, ensuring that only relevant features are passed to the decoding layers. We evaluate the robustness of the proposed method across facial, medical, and remote sensing domains. The experimental results demonstrate that HREDN excels in preserving edge details and structural features in denoised images, outperforming state-of-the-art techniques in both qualitative and quantitative measures. Statistical analysis further highlights the model’s ability to effectively remove noise in diverse, complex scenarios with images of varying resolutions across multiple domains.

## 1. Introduction

Image denoising is a critical aspect of image processing, focusing on the restoration of the original image through the reduction of noise in a noisy version. It aids in resolving additional image processing issues and is categorized into two primary methods, traditional filtering and deep learning [1,2,3,4,5,6]. The traditional filtering approach involves employing mathematical operations and spatial filters like mean or median filters, but its effectiveness is limited when confronted with complex situations. Deep learning leverages CNNs to acquire knowledge of noise patterns through pairs of noisy and clean images, leading to enhanced denoising with higher accuracy. Image denoising holds significant importance in the field of research and has the capacity to improve diverse image processing applications.

Traditional filters, such as Gaussian filter, median filter, bilateral filter, and BM3D, have been widely employed, but they do not achieve state-of-the-art denoising performance [1,2,3,4].

The availability of vast amounts of data and high processing power has led to the development of several neural network-based techniques for image processing. A CNN model was proposed by Gose et al. for image denoising [7]. It achieved optimal metric scores compared to traditional filtering methods; however, the authors did not compare the proposed technique with current state-of-the-art methods, and the images were noised with limited noise levels. Ronneberger et al. proposed the U-Net architecture, which is an encoder–decoder-based model featuring a skip connection between the encoding and decoding layers [8]. This innovative design enabled selective data transfer, leading to improved image reconstruction. Oktay et al. introduced a novel attention gate (AG) model that automatically directs attention to target structures of varying sizes and shapes, eliminating the need for external localization modules [9]. This innovation enhances the accuracy of CNN architectures like U-Net without introducing significant computational overhead. The experimental results demonstrate that AGs enhance U-Net’s performance across various datasets and training sizes while maintaining computational efficiency. In their work, Zhang et al. introduced the application of feed-forward denoising convolutional neural networks [10]. They harness advancements in deep architecture, learning algorithms, and regularization techniques to address image denoising. To expedite the training process, the incorporation of residual learning and batch normalization has been employed, thereby resulting in an augmentation of the denoising performance. Cha et al. implemented a novel fully convolutional architecture, which greatly improves the base supervised model and introduces regularization methods for adaptive fine-tuning [11]. This results in a more potent and robust adaptivity. In their study, Tian et al. introduced an image denoising method based on an attention-guided denoising convolutional neural network [12]. This network comprises several components, namely a sparse block, a feature enhancement block, an attention block, and a reconstruction block. Tian et al. proposed a novel network called a batch renormalization denoising network (BRDNet) [13]. To obtain more features, the width of the network was increased, and batch renormalization was also used. Li et al. introduced a novel Gated Fully Fusion (GFF) architecture for semantic segmentation, where gates are employed in a fully connected manner to fuse features across multiple levels [14]. The proposed method exhibited state-of-the-art performance on four challenging scene parsing datasets. Gurrola-Ramos et al. introduced a residual dense neural network (RDUNet) for image denoising [15]. This encoder–decoder-based architecture features densely connected convolutional layers that enable local residual learning and the reuse of feature maps. The proposed method achieved competitive results without requiring prior knowledge of noise levels. In their work, Zhang et al. developed RatUNet, a refined deep convolutional U-Net architecture aimed at image denoising [16]. It enhances skip connections, downsampling, upsampling, and network depth, while also incorporating polarized and depthwise self-attention mechanisms. Mafi et al. introduce a deep convolutional neural network that combines batch normalization and regularization to effectively remove mixed Gaussian and impulse noise [17]. Their model demonstrates optimal structural metric performance when addressing both familiar and unfamiliar noise combinations. Khmag introduced a self-adjusting generative confrontation network designed for denoising digital images [18]. The method demonstrated quantitative performance and visual quality on par with machine learning approaches such as DnCNN and SuKro, while also offering faster processing speeds [10,19]. Khmag integrated second-generation wavelet transformation with a convolutional neural network to enhance the deblurring of digital images [20]. The proposed approach achieved superior results compared to BM3D, MLP, SSDA, and traditional neural networks [21,22,23]. Cheng et al. introduced a multi-domain encoder–decoder latent data assimilation (MEDLA) framework for dynamical systems [24]. This approach effectively reduces computational burden while enhancing assimilation accuracy. Cheng et al. proposed a novel multi-scale physics-constrained neural network (MSPCNN) for dynamical systems [25]. This innovative approach demonstrates noise robustness and enhances prediction accuracy when physical constraints are introduced in low-fidelity fields. Chen et al. introduced TransUNet, which combines Transformers with UNET to offer a dependable and efficient method for medical image segmentation [26]. The global self-attention mechanism of Transformers, integrated into UNET-based architecture, could inspire advancements in image denoising.

Although CNNs have become widely favored in the field of image processing, their performance tends to deteriorate as plain CNN architecture deepens, limiting their capacity for feature extraction. Some networks fail to effectively share features between shallow and deep layers. Additionally, many networks overlook edge information, leading to poor structural similarity between the denoised image and the original image.

In this paper, we propose a highly robust encoder–decoder architecture, HRED, which integrates a multi-scale feature extraction block and an attention gate to address key challenges in image denoising. The framework employs an encoder–decoder architecture where downsampling extracts feature maps, and upsampling reconstructs a clean image. However, encoding can result in loss of information, reducing the decoder’s ability to preserve structural details. To mitigate this, skip connections transfer feature map information from the encoder to the decoder [8]. While effective, skip connections may also propagate redundant information from encoding to decoding layers. To address this, we incorporate attention gates within the skip connections, ensuring only relevant features are passed forward. Additionally, skip connections help to mitigate the vanishing gradient problem in deep networks [27]. To further enhance feature extraction capabilities, we integrate a multi-scale feature enhancement block into the encoder. This block captures features at varying scales, improving the network’s capacity to handle complex noise patterns. The experimental results demonstrate that HRED surpasses state-of-the-art denoising methods across multiple datasets, achieving superior MSE, PSNR, SSIM, and IEF values [28,29]. HREDN can be used as a preprocessing step in facial image analysis, medical imaging, and remote sensing. Images in these fields are particularly prone to various types of noise. As a result, HREDN effectively removes noise and enhances image clarity, which in turn improves accuracy in tasks such as segmentation, classification, object detection, and more.

Although BM3D is highly effective at removing noise when the variation in an image is continuous, such as Gaussian noise, the addition of salt-and-pepper noise increases the sparsity of the noise, introducing random extreme values. This reduces the efficacy of BM3D, as clearly demonstrated in the results. Therefore, a convolutional neural network-based method offers a more practical solution for this scenario. DNCNN, FCAIDE, and BRDNET are well-known convolutional neural networks commonly used for image denoising. However, they all lack an effective attention mechanism, which can lead to the propagation of superfluous information into the deeper layers of the network. Additionally, these models do not include mechanisms such as multi-scale feature enhancement blocks, which are crucial for effectively handling complex noise. Although the Gated Fully Fusion (GFF) network facilitates the propagation of useful information from the encoder to the decoder, potentially reducing noise, the experimental results suggest that it is less effective than the attention gate. RDUNET, on the other hand, adopts an encoder–decoder architecture like HREDN, where information is transferred from the encoder to the decoder through skip connections. However, it does not incorporate attention gates within these skip connections, which are vital for ensuring that only relevant information is passed to the decoder. Furthermore, RDUNET lacks specific mechanisms for multi-scale feature extraction, limiting its ability to address complex noise effectively. In contrast, TransUNET and SwinUNET represent advanced Transformer-based architecture. These models leverage self-attention mechanisms to capture global context effectively. However, they lack an inherent capability to focus on spatially local details. Additionally, Transformer-based models are computationally intensive, making them challenging to train in resource-constrained environments. As a result, in this research, we employed two different Transformer-based models for facial, CT-scan, and remote sensing image denoising to achieve optimal results. Lastly, the HREDN model draws inspiration from both U-Net and Attention U-Net. It improves upon these designs by introducing advanced attention mechanisms and incorporating multi-scale feature extraction capabilities, making it particularly well-suited for denoising complex noise.

## 2. Materials and Methods

### 2.1. Image Noise

This research primarily focuses on Gaussian noise and salt-and-pepper noise. Gaussian noise occurs when an image is distorted by the introduction of a random Gaussian function. Meanwhile, salt-and-pepper noise is generated when bright and dark spots are randomly inserted into an image. Mixed noise arises when an image undergoes distortion caused by a combination of multiple noise types [17].

Mathematically, the process of adding mixed noise (Gaussian + salt-and-pepper) in an image is shown below:

(1) $$I_{n}=f_{s\&p}\left(I_{c}+w\times G\right)$$

where $I_{n}$ denotes a noised image, while $I_{c}$ corresponds to its noise-free original image. The presence of Gaussian noise is represented by the variable G, with its noise factor denoted by w. Additionally, the function $f_{s\&p}$ represents a function that randomly add salt-and-pepper noise to the image.

**Figure 1:**
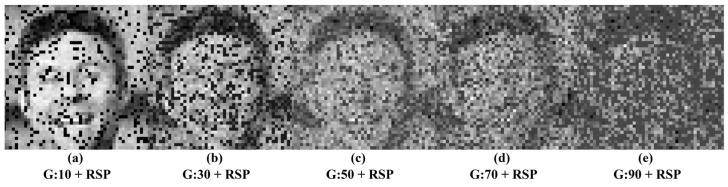
Images corrupted by mixed Gaussian (G) and random salt-and-pepper (RSP) noise: (**a**) G:10 + RSP, (**b**) G:30 + RSP, (**c**) G:50 + RSP, (**d**) G:70 + RSP, (**e**) G:90 + RSP.

In this study, images corrupted by five distinct types of mixed noise are used. An example of an image corrupted by these five noise types is shown in Figure 1.

### 2.2. Network Architecture

**Figure 2:**
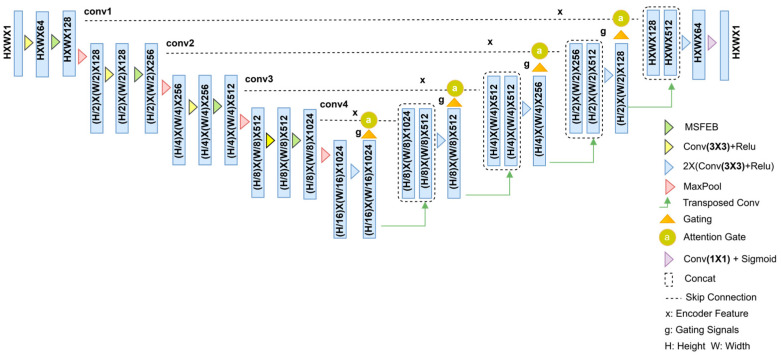
Architecture of the proposed HREDN.

In this paper, we propose a highly robust encoder–decoder network (HREDN). The structure of the proposed network is illustrated in Figure 2.

As shown in Figure 2, our network adopts an encoder–decoder structure, where the encoder consists of four encoding modules. Each module includes four 3 × 3 convolutional layers, four multi-scale feature enhancement blocks, and four max-pooling layers with a stride of 2 × 2. Two 3 × 3 convolutional layers are included in the intermediate stage.

Meanwhile, the decoder consists of four 3 × 3 transposed convolutional layers with a stride of 2 × 2 and four pairs of 3 × 3 convolutional layers. At the end of the network, a 1 × 1 convolutional layer with a sigmoid activation function is added. In addition to this, ReLU is used as the activation function in all other convolutional layers to enhance the network’s nonlinearity representation [30].

#### 2.2.1. Residual Encoder–Decoder Architecture

We use a residual encoder–decoder architecture as the backbone of our HREDN. When passing an image through the encoder block, there is a risk of information loss, which reduces the decoder’s capacity to retain structural details. To address this issue, skip connections are employed to transfer information from the feature maps of the encoder to the decoder.

The encoder–decoder architecture is symmetric, so skip connections can only be added between encoder and decoder layers at corresponding levels. However, skip connections can also transfer superfluous information from encoding layers to decoding layers. To prevent this, an attention gate is introduced within the skip connection. Additionally, in deep networks, skip connections help address the vanishing gradient problem [27].

#### 2.2.2. Multi-Scale Feature Enhancement Block (MSFEB)

**Figure 3:**
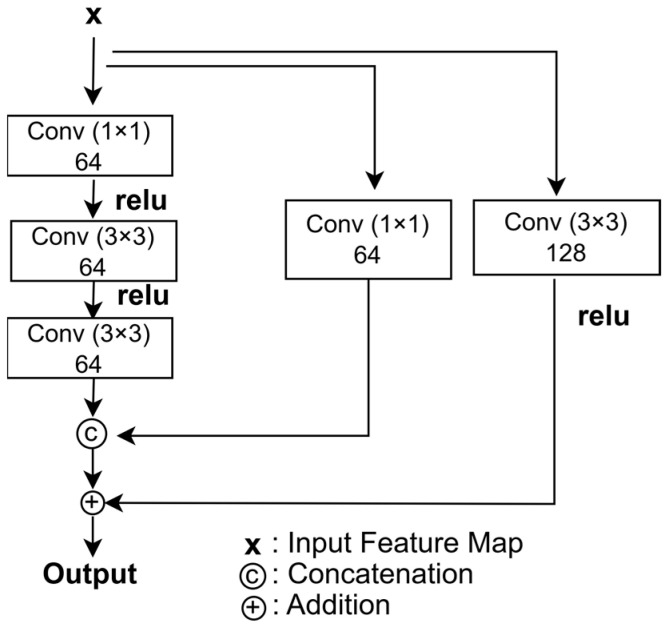
Architecture of the multi-scale feature enhancement block.

In this paper, a multi-scale feature enhancement block is constructed to extract features at varying scales. The multi-scale feature enhancement block designed in this paper is shown in Figure 3.

Convolutional layers with varying filter sizes and numbers are combined to enhance the network’s feature extraction capability. ReLU is applied in the convolutional layers to maintain nonlinearity. Additionally, a 1 × 1 convolutional kernel is used to reduce computational overhead.

Learning features at multiple scales allows the model to capture both fine details and broader patterns in the data. Varying the number of filters enables the network to detect more complex patterns, improving its performance in handling intricate noise. This approach boosts the model’s robustness and enhances its ability to represent diverse features.

#### 2.2.3. Attention Gate

**Figure 4:**
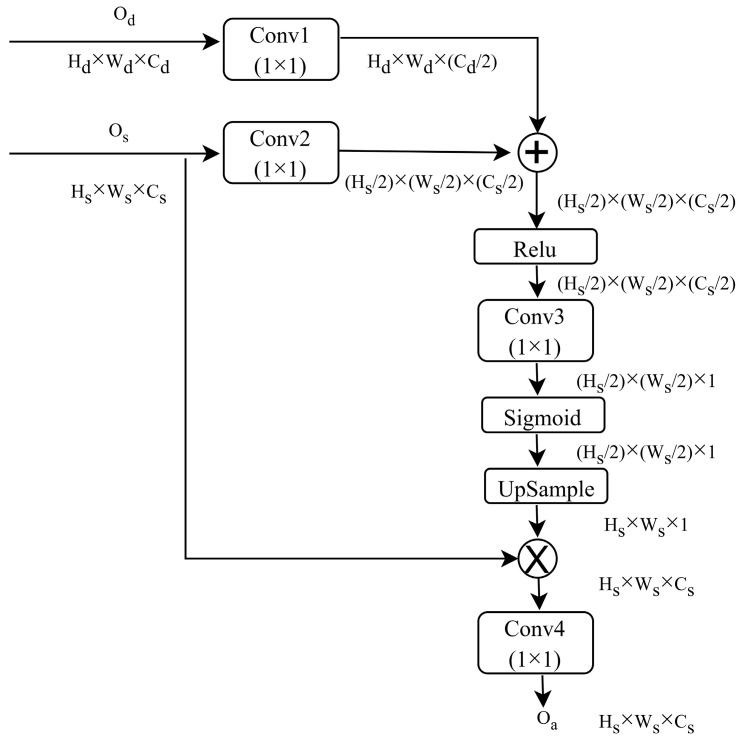
In-depth architecture of proposed attention block.

The attention gate in HREDN is inspired by the additive attention gate [9]. This attention gate is integrated into UNET architecture to enhance its performance. It selectively focuses on significant activations, enabling the extraction of the most relevant data features from the information passed through the skip connection. As a result, irrelevant and noisy responses are filtered out, leading to improved performance. The attention process occurs just before the concatenation operation, ensuring that only pertinent activations are combined. The proposed architecture of the attention block is shown in Figure 4.

As shown in Figure 4, two inputs are passed to the attention block. The input named $O_{s}$ is generated from the upper layer, producing a representation with higher dimensions, whereas $O_{d}$ is generated from the lower layer, resulting in a representation with lower dimensions. To maintain uniform dimensions, a relevant convolution operation is applied, and the resulting representations are then added.

The process of aligning the dimensions of two input vectors followed by the concatenation of these aligned inputs is as shown in Equation (2).

(2) $$O_{ds}=R1\left(Conv\left(O_{d}\right)\right)⊕R2\left(Conv\left(O_{s}\right)\right)$$

where $O_{s}$ represents the value gained from skip connection, $O_{d}$ represents the output of decoder, and $O_{ds}$ represents the output gained after addition. Conv is a 1 × 1 convolution operator, and R1 and R2 are tensor reshape operators.

Afterward, a rectified linear unit (ReLU) is employed as an activation function, and the resulting tensor undergoes a convolutional layer with a filter count of 1. This produces a single-depth vector, representing the input’s weight. To maintain interpretability, the gained output undergoes additional handling through a sigmoid activation function.

The process of the gaining weight vector is shown in Equation (3).

(3) $$w=Sigmoid\left(R3\left(Conv\left(Relu\left(O_{ds}\right)\right)\right)\right)$$

where R3 is the tensor reshape operator, Sigmoid represents sigmoid activation function, w represents gained weight vector, Conv is a 1 × 1 convolution operator, and Relu represents the rectified linear unit activation function.

The resulting weight vector is upsampled to match the dimensions of $O_{s}$. The vector $O_{s}$ and weight vector undergo element-wise multiplication, which scales the elements according to their relevance, effectively adjusting the vector. Finally, the output gained after multiplication is convolved and passed to the decoder.

The process of adjusting the $O_{s}$ with respect to the weight vector is shown in Equation (4).

(4) $$O_{a}=R4\left(Conv\left(O_{s}⊗Upsample\left(w\right)\right)\right)$$

where R4 is tensor reshape operator, Conv is a 1 × 1 convolution operator, and Upsample is the upsampling layer.

### 2.3. Loss Function

To enhance the performance of the HREDN model, its parameters need to be optimized. This can be achieved by minimizing the loss function during the model training process. Equation (5) represents the loss function used in this research.

(5) $$L\left(\theta \right)=\frac{1}{K}\sum_{i=1}^{K}{\left(f_{HREDN}\left(I_{N}\left(i\right),\theta \right)-I_{c}\left(i\right)\right)}^{2}$$

where K is the number of noisy images, θ denotes the parameters of HREDN, $I_{N}\left(i\right)$ is ith noisy image, and $I_{c}\left(i\right)$ is ith clean image.

### 2.4. Quantitative Evaluation

To quantitatively evaluate the performance of the proposed models, mean square error (MSE), peak signal-to-noise ratio (PSNR) [28], structural similarity (SSIM) [29], and image enhancement factor (IEF) are used. They can be defined as follows:

(6) $$MSE=\frac{1}{n}\sum_{i=1}^{n}{\left(I_{denoised,\;i}-I_{original,i}\right)}^{2}$$

where $I_{denoised,\;i}$ represents the denoised image at pixel i, $I_{original,i}$ represents the original noise-free image at pixel i, and n denotes the total number of pixels in the image.

(7) $$PSNR\left(dB\right)=10{log}_{10}\left[{MAX}_{Signal}^{2}/MSE\right]$$

where MSE represents mean square error of all the pixels in the images and MAX represents the maximum value of pixel.

(8) $$S\left(x,y\right)=\left[\frac{2\mu_{x}\mu_{y}+C_{1}}{\mu_{x}^{2}+\mu_{y}^{2}+C_{1}}\right]\cdot \left[\frac{2\sigma_{x}\sigma_{y}+C_{2}}{\sigma_{x}^{2}+\sigma_{y}^{2}+C_{2}}\right]\cdot \left[\frac{\sigma_{xy}+C_{3}}{\sigma_{x}\sigma_{y}+C_{3}}\right]$$

where μ denotes the means, σ denotes the variance, C denotes the constant, and $\sigma_{xy}$ denotes the covariance.

(9) $$IEF=\frac{\sum {\left(I_{noisy}-I_{original}\right)}^{2}}{\sum {\left(I_{denoised}-I_{original}\right)}^{2}}$$

where $I_{original}$ represents the original image, $I_{noisy}$ represents the noisy image, and $I_{denoised}$ represents the denoised image.

## 3. Results

### 3.1. Dataset

To train our model for mixed Gaussian and salt-and-pepper noise denoising, our training set is constructed by using images from the Facial Expression Recognition 2013 Dataset (FER2013) [31]. The FER2013 dataset consists of 35,887 grayscale images, each with a resolution of 48 × 48 pixels. To train, validate, and test the deep learning model, 25,120 images (70%), 5383 images (15%), and 5384 images (15%) are designated, respectively. Furthermore, for additional testing, the CKPLUS-48 dataset is utilized, which comprises 750 grayscale images, that also have a resolution of 48 × 48 pixels [32]. The deep learning model trained on the FER2013 dataset is employed to denoise the noisy CKPLUS images.

To diversify the dataset, the Curated COVID CT dataset is used [33]. It includes 17,104 grayscale CT images of 128 × 128 pixels, divided into 70% for training, 15% for validation, and 15% for testing.

To further evaluate the model, the NWPU-RESISC45 dataset is used [34]. This remote sensing image dataset contains 31,500 images spanning 45 scene classes, with 700 images per class. A subset of 17,500 grayscale images from 25 scene classes (700 images per class) is selected. Each image has a resolution of 128 × 128 pixels. The dataset is split into 70% for training, 15% for validation, and 15% for testing.

### 3.2. Mixed Noise Generation

To generate noisy images, a mixture of Gaussian and salt-and-pepper noise is used. Gaussian noise is applied with noise factors of 10, 30, 50, 70, and 90. Meanwhile, salt-and-pepper noise is randomly generated for each image in the dataset, with a replacement range of 1% to 50%. The range of salt-and-pepper noise is limited compared to Gaussian noise because the impact of small amounts of salt-and-pepper noise is equivalent to that of high-value Gaussian noise. This ensures that neither type of noise has a disproportionate influence.

As shown in Table 1, Gaussian noise with a noise factor of 30 and salt-and-pepper noise with 5% replacement yield similar metric values.

### 3.3. Experiment Settings

We used the Adam optimizer with $\beta_{1}=0.9,\;\;\beta_{2}=0.999$, and $\varepsilon =10^{-7}$ to minimize the loss function for the training model [35]. The learning rate is 0.001, the number of training epochs is 100, and the batch size is 64. Early stopping with a patience value of 2 is used to monitor the validation loss and prevent overfitting. For CT scan and remote sensing image denoising, a batch size of 16 is used.

We use TensorFlow version 2.1.0, keras version 2.3.1, and python version 3.7.6 to train and test models, and all experiments were run on a Kaggle with NVIDIA T4(×2) GPU.

### 3.4. Analysis of Results

This section shows the experimental results. For the facial image denoising, the experimental result of the HREDN is compared with several recent state-of-the-art methods, such as BM3D, DNCNN, FCAIDE, ADNET, BRDNET, GFF, RDUNET, and TransUNET [4,10,11,12,13,14,15,26]. For CT scan image denoising, SwinUNet is used instead of TransUNet [36]. TransUNet is computationally more expensive and challenging to train with the current setup, whereas SwinUNet offers more efficient memory usage.

Among the compared methods, BM3D is a representative model-based method, while SwinUNET and TransUNET are CNN-Transformer-based models, and the remaining methods are CNN-based denoising techniques. All models are re-implementations of the original training processes.

#### 3.4.1. Facial Image Dataset

**Figure 5:**
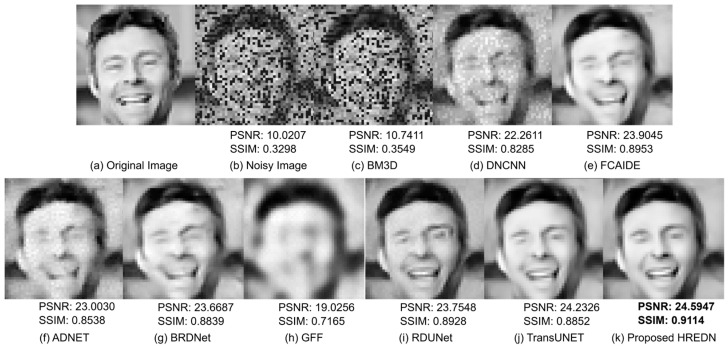
Visual comparison among eight facial image denoising methods on a single testing image from the FER2013 dataset with noise level (G:30 + RSP).

Figure 5 presents the qualitative results of each model after denoising a facial image corrupted by a mixture of Gaussian noise with a noise factor of 30 and random salt-and-pepper noise. From the figure, it is evident that BM3D fails to remove the noise completely. Noise remains clearly visible in the image produced by DNCNN as well. The denoised image produced by the GFF is blurry, and the facial details are unclear. While ADNET and BRDNET successfully remove the noise, these methods fail to preserve structural details. RDUNET, FCAIDE, RDUNET, and TransUNET generate smoother and clearer images; however, the proposed HREDN demonstrates superior performance in preserving edge information and structural details. This is particularly noticeable in the edges of the cheek, where HREDN effectively retains the facial structure.

Table 2, Table 3, Table 4 and Table 5 present the metric values for each model after denoising the facial image. From the table, it is evident that the proposed HREDN achieves the lowest MSE, indicating that the denoised image closely matches the original in terms of pixel values. Additionally, HREDN exhibits the highest PSNR and SSIM scores, suggesting that it effectively removes noise while maintaining high image quality and preserving visual similarity to the original. HREDN also preserves key structural details. Finally, HREDN achieves the highest IEF score, demonstrating its ability to enhance edges and textures without introducing artifacts.

In addition to the FER2013 dataset, the performance of the proposed HREDN is also compared with state-of-the-art algorithms on the CKPLUS dataset. The CKPLUS dataset, which is another facial image dataset, is used for denoising tasks. Since the FER2013 dataset consists of facial images, using a denoising dataset with similar image types is a logical choice. Models trained on the FER2013 dataset are applied to denoise noisy images from the CKPLUS dataset.

**Figure 6:**
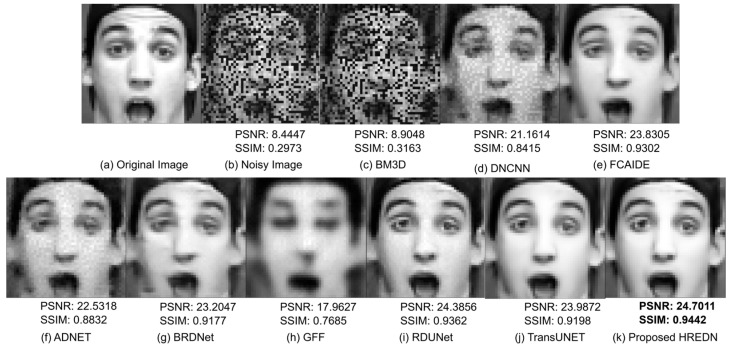
Visual comparison results for eight facial image denoising methods on single testing image from CKPLUS dataset with noise level (G:30 + RSP).

Figure 6 shows the qualitative results of each model after denoising a facial image corrupted by a mixture of Gaussian noise with a noise factor of 30 and random salt-and-pepper noise. The figure clearly demonstrates that the image generated by BM3D is largely unable to remove noise. Noise remains visible in the images produced by DNCNN and ADNET. The denoised image produced by the GFF is blurry, and the facial features are not visible. The images generated by BRDNET and FCAIDE appear blurry. RDUNET and TransUNET successfully produce clear, noise-free images, but the edge information and structural details are better preserved in the image generated by HREDN. HREDN effectively reduces noise while maintaining finer details, such as facial features and the overall shape of the head.

Table 6, Table 7, Table 8 and Table 9 present the denoising performance of each model. HREDN stands out with the lowest MSE and highest PSNR, indicating superior noise reduction and image quality. It also excels in preserving structural details and enhancing edges, as evidenced by its highest SSIM and IEF scores.

#### 3.4.2. CT Scan Dataset

To further evaluate the performance of HREDN in other domains, it is applied to denoise CT scan images.

**Figure 7:**
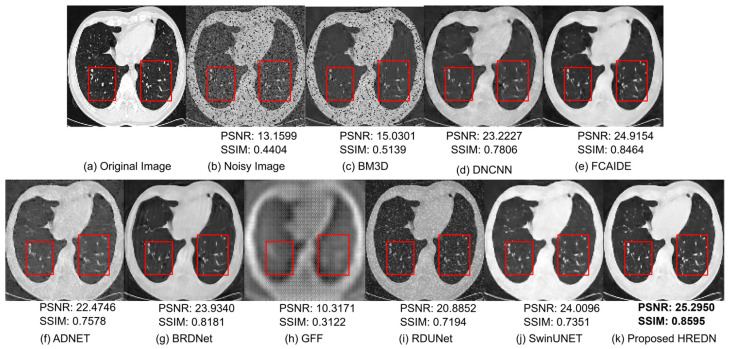
Visual comparison results for eight CT scan image denoising methods on single testing image from Curated COVID CT dataset with noise level (G:30 + RSP).

Figure 7 shows the qualitative results of each model after denoising a CT scan image corrupted by a mixture of Gaussian noise (with a noise factor of 30) and random salt-and-pepper noise. To better observe the denoising performance of each model, a red-boxed region of interest is highlighted in the image. The figure clearly demonstrates that the image generated by BM3D fails to remove noise effectively. Noise remains visible in the image produced by RDUNET. The region of interest is blurry in the case of GFF, and the model is unable to preserve crucial details. Meanwhile, DNCNN, ADNET, and BRDNET struggle to preserve edge information in the region of interest. Although the images generated by FCAIDE and SwinUNET appear clearer, the structural information is better preserved in the image produced by the proposed HREDN. Additionally, the edge information in the HREDN-generated image is notably sharper.

Table 10, Table 11, Table 12 and Table 13 present the metric values for each model after denoising the CT scan images. From the table, it is evident that the proposed HREDN achieves the lowest MSE and the highest PSNR, SSIM, and IEF values. The proposed HREDN outperforms other state-of-the-art methods by a significant margin in nearly all mixed noise cases, achieving optimal metric scores.

#### 3.4.3. NWPU-RESISC45 Dataset

To further evaluate the performance of HREDN in other domains, it is applied to denoise remote sensing images.

**Figure 8:**
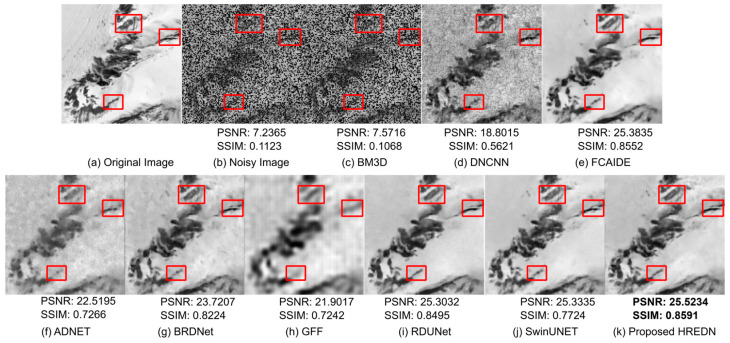
Visual comparison results for eight remote sensing image denoising methods on single testing image from NWPU-RESISC45 dataset with noise level (G:30 + RSP).

Figure 8 illustrates the qualitative results of each model after denoising a mountain image affected by a combination of Gaussian noise (with a noise factor of 30) and random salt-and-pepper noise. To enhance the evaluation of denoising performance, a red-boxed region of interest is highlighted in the image. The figure clearly demonstrates that the BM3D algorithm struggles to effectively remove noise. The denoised image produced by DNCNN still contains noticeable noise, while the region of interest appears blurry in the image processed by GFF. Although ADNET performs better than DNCNN, noise remains, and the region of interest lacks clarity. BRDNet significantly reduces noise, but the region of interest is still not well-defined. FCAIDE, RDUNet, and SwinUNET successfully remove noise while preserving edge information; however, the proposed HREDN outperforms them in retaining structural details. This is evident from both the obtained metric scores and the visualization of the region of interest.

Table 14, Table 15, Table 16 and Table 17 display the metric values for each model following the denoising of remote sensing images. The data clearly indicate that the proposed HREDN attains the lowest MSE and the highest PSNR, SSIM, and IEF values. In nearly all mixed noise scenarios, HREDN surpasses other state-of-the-art methods by a considerable margin, achieving superior metric scores.

#### 3.4.4. Statistical Evaluation of HREDN’s Generalization Across Domains

To evaluate the robustness of HREDN across all domains, a statistical analysis was conducted. For the facial domain, only the FER2013 dataset results were used, as it was directly used for training the model. An ANOVA test was performed to determine whether HREDN’s mean performance differed across domains, while Levene’s test was conducted to assess the similarity of variances across groups. Together, these tests provide a statistically rigorous generalization analysis.

As shown in Table 18, the ANOVA results indicate that there are no statistically significant differences across domains for PSNR (*p* = 0.1585), SSIM (*p* = 0.3641), and IEF (*p* = 0.1585), suggesting that the model performed consistently. While MSE showed some variation (*p* = 0.0963), it did not reach statistical significance. Additionally, Levene’s test confirmed that the variance across domains was homogeneous for all metrics (*p* > 0.05). These findings suggest that the model generalizes well across different image domains.

Compared to the medical and remote sensing domains, the facial images are significantly smaller, resulting in fewer pixels, each carrying more information. As a result, noise has a greater impact, leading to higher MSE variation in the facial domain. Nonetheless, the model effectively handles this with only small performance degradation.

### 3.5. Ablation Studies

Ablation experiments are conducted to assess the contribution of each module in the proposed network architecture. The experiments are performed under mixed noise conditions (G:30 + RSP) using the FER2013 dataset for facial image denoising.

- ED: A basic encoder–decoder architecture with a skip connection.
- ED + Attention: A basic encoder–decoder architecture with a skip connection enhanced by an attention gate.
- ED + Attention + MSFEB: A basic encoder–decoder architecture with a skip connection enhanced by an attention gate and a multi-scale feature enhancement block.

Table 19 presents the average performance metric scores obtained by integrating the attention gate and MSFEB module into an encoder–decoder architecture. The attention gate improves the extraction of relevant features, leading to the enhanced performance of the model. Furthermore, the inclusion of multi-scale features through the MSFEB module results in a significant and more pronounced improvement in model performance. This underscores the crucial role of edge and structural information captured by MSFEB in enhancing the overall effectiveness of the network.

Table 20 presents a summary of the training time, number of parameters, and inference time gained after integrating each module into the basic encoder–decoder architecture. The values for each performance metric shown in the table represent averages derived from all mixed noise cases. It is evident from the table that incorporating attention mechanisms has led to a reduction in both training and inference times. This suggests that the model prioritizes relevant input features while disregarding unnecessary information, though at the cost of an increased number of parameters. Additionally, the inclusion of MSFEB has resulted in a significant increase in all performance metrics, indicating a higher requirement for computational time and resources during model training.

The impact of adding attention and MSFEB modules is analyzed in Table 19 and Table 20 to understand their contributions to model performance and computational efficiency. While the addition of these modules has improved the model’s performance, it comes at the cost of increased computational burden. The main objective of this study is to denoise digital images in complex noise scenarios, which requires a sophisticated network architecture capable of capturing intricate noise patterns. However, for tasks involving denoising images with lower complexity, the model can be simplified.

## 4. Conclusions

In summary, a novel and highly robust encoder–decoder network (HREDN) for denoising mixed salt-and-pepper and Gaussian noise is proposed. HREDN uses a multi-scale feature enhancement block in the encoder to capture features at various scales. Skip connections transfer important feature maps from the encoder to the decoder to preserve structural details, while attention gate ensures only relevant features are passed, eliminating redundant information. The experimental results show that the proposed method significantly outperforms existing approaches across all types of Gaussian and salt-and-pepper noise mixtures in facial, medical, and remote sensing image denoising. The method effectively preserves edge information and structural details in the denoised images, achieving superior performance in both qualitative and quantitative metrics compared to existing methods. The statistical analysis also demonstrates the model’s robustness in removing noise across multiple complex noise scenarios, with varying image resolutions across different domains.

In future work, we plan to investigate more advanced deep learning architectures and expand the proposed network to other domains. Additionally, we aim to explore a wider range of noise scenarios beyond mixed Gaussian and salt-and-pepper noise.

## Figures and Tables

**Table 1:** PSNR and SSIM value gained after adding noise.

Original vs. Noisy Image		
Noise Type	PSNR	SSIM
Gaussian (30)	18.59	0.56
Salt-and-Pepper (5%)	18.45	0.62

**Table 2:** Average MSE gained before and after denoising 5384 Fer2013 testing images.

Methods	G:10 + RSP	G:30 + RSP	G:50 + RSP	G:70 + RSP	G:90 + RSP
Original vs. Noisy					
	0.0839	0.0932	0.1115	0.1389	0.1754
Original vs. Denoised					
BM3D	0.0684 ± 0.059	0.0755 ± 0.060	0.0942 ± 0.059	0.1233 ± 0.054	0.1626 ± 0.049
DNCNN	0.0031 ± 0.002	0.0051 ± 0.002	0.0111 ± 0.014	0.0109 ± 0.003	0.0187 ± 0.005
FCAIDE	0.0011 ± 0.001	0.0031 ± 0.001	0.0052 ± 0.002	0.0073 ± 0.002	0.0087 ± 0.003
ADNET	0.0027 ± 0.002	0.0041 ± 0.001	0.0075 ± 0.003	0.0095 ± 0.003	0.0121 ± 0.004
BRDNet	0.0011 ± 0.001	0.0034 ± 0.001	0.0058 ± 0.002	0.0075 ± 0.003	0.0102 ± 0.003
GFF	0.0119 ± 0.023	0.0110 ± 0.009	0.0153 ± 0.035	0.0163 ± 0.060	0.0134 ± 0.005
RDUNET	0.0015 ± 0.001	0.0031 ± 0.001	0.0053 ± 0.002	0.0071 ± 0.002	0.0176 ± 0.006
TransUNET	0.0012 ± 0.001	0.0029 ± 0.001	0.0054 ± 0.002	0.0068 ± 0.002	0.0090 ± 0.003
HREDN	**0.0009 ± 0.001**	**0.0027 ± 0.001**	**0.0047 ± 0.002**	**0.0066 ± 0.002**	**0.0085 ± 0.003**

**Table 3:** Average PSNR gained before and after denoising 5384 Fer2013 testing images.

Methods	G:10 + RSP	G:30 + RSP	G:50 + RSP	G:70 + RSP	G:90 + RSP
Original vs. Noisy					
	12.1564	11.2294	10.0542	8.8551	7.7067
Original vs. Denoised					
BM3D	14.0295 ± 55.47	13.1275 ± 4.78	11.1517 ± 2.91	9.4634 ± 1.78	8.0671 ± 1.22
DNCNN	26.0204 ± 2.72	23.1781 ± 1.54	20.6251 ± 2.46	19.7788 ± 1.14	17.4133 ± 1.07
FCAIDE	29.8812 ± 1.96	5.3660 ± 1.41	23.0189 ± 1.41	21.5827 ± 1.40	20.8224 ± 1.45
ADNET	26.7243 ± 2.94	24.0540 ± 11.32	21.5050 ± 1.46	20.4072 ± 1.30	19.3431 ± 1.26
BRDNet	30.1462 ± 1.96	24.9166 ± 1.40	22.5217 ± 1.28	21.4418 ± 1.38	20.1332 ± 1.32
GFF	20.9552 ± 3.03	20.1160 ± 1.95	19.3942 ± 2.39	19.1173 ± 2.08	18.9849 ± 1.48
RDUNET	28.4322 ± 1.22	25.2940 ± 1.51	23.0055 ± 1.43	21.7566 ± 1.47	17.7947 ± 1.41
TransUNET	29.6051 ± 1.93	25.5655 ± 1.48	22.8748 ± 1.35	21.9385 ± 1.43	20.6780 ± 1.38
HREDN	**30.6853 ± 1.92**	**25.8967 ± 1.52**	**23.5542 ± 1.48**	**22.0414 ± 1.53**	**20.9744 ± 1.50**

**Table 4:** Average SSIM gained before and after denoising 5384 Fer2013 testing images.

Methods	G:10 + RSP	G:30 + RSP	G:50 + RSP	G:70 + RSP	G:90 + RSP
Original vs. Noisy					
	0.3554	0.2895	0.2253	0.1746	0.1364
Original vs. Denoised					
BM3D	0.4253 ± 0.22	0.3717 ± 0.20	0.2503 ± 0.11	0.1746 ± 0.07	0.1308 ± 0.06
DNCNN	0.8763 ± 0.07	0.7800 ± 0.07	0.7202 ± 0.06	0.6540 ± 0.06	0.4632 ± 0.07
FCAIDE	0.9581 ± 0.02	0.8833 ± 0.03	0.8128 ± 0.04	0.7589 ± 0.05	0.7123 ± 0.06
ADNET	0.9060 ± 0.05	0.8282 ± 0.04	0.7323 ± 0.07	0.6827 ± 0.05	0.6134 ± 0.05
BRDNet	0.9585 ± 0.02	0.8681 ± 0.03	0.7892 ± 0.04	0.7392 ± 0.05	0.6778 ± 0.05
GFF	0.7303 ± 0.10	0.7169 ± 0.06	0.6828 ± 0.07	0.6468 ± 0.08	0.6323 ± 0.07
RDUNET	0.9334 ± 0.02	0.8860 ± 0.03	0.8159 ± 0.04	0.7692 ± 0.05	0.5760 ± 0.06
TransUNET	0.9394 ± 0.02	0.8483 ± 0.05	0.7318 ± 0.06	0.7099 ± 0.07	0.6337 ± 0.08
HREDN	**0.9632 ± 0.01**	**0.8951 ± 0.03**	**0.8330 ± 0.04**	**0.7759 ± 0.06**	**0.7336 ± 0.06**

**Table 5:** Average IEF gained before and after denoising 5384 Fer2013 testing images.

Methods	G:10 + RSP	G:30 + RSP	G:50 + RSP	G:70 + RSP	G:90 + RSP
Original vs. Denoised					
BM3D	11.0443 ± 661.91	3.2545 ± 105.03	1.3066 ± 0.38	1.1426 ± 0.08	1.0856 ± 0.03
DNCNN	26.8849 ± 10.07	17.0285 ± 7.02	14.1173 ± 7.83	12.8376 ± 4.02	9.5817 ± 2.13
FCAIDE	72.3963 ± 43.81	30.2600 ± 17.76	21.8154 ± 11.78	19.8386 ± 9.01	21.7623 ± 9.25
ADNET	31.0252 ± 10.78	22.6659 ± 13.47	15.7620 ± 8.18	15.1775 ± 5.85	15.2315 ± 4.75
BRDNet	76.4292 ± 45.00	26.9473 ± 15.02	19.4890 ± 9.62	19.2113 ± 7.75	19.2113 ± 7.75
GFF	9.9257 ± 6.65	9.1453 ± 5.26	9.5543 ± 4.19	11.5889 ± 4.92	14.2739 ± 5.36
RDUNET	53.2546 ± 32.30	29.6204 ± 17.28	21.7541 ± 10.68	20.7435 ± 8.76	10.6554 ± 3.19
TransUNET	67.4204 ± 39.47	32.1680 ± 0.20	21.1724 ± 10.72	21.5955 ± 8.76	21.0163 ± 8.26
HREDN	**88.6590 ± 58.44**	**34.6337 ± 21.51**	**25.0132 ± 14.07**	**22.5668 ± 18.92**	**22.7099 ± 13.77**

**Table 6:** Average MSE gained before and after denoising CKPLUS images.

Methods	G:10 + RSP	G:30 + RSP	G:50 + RSP	G:70 + RSP	G:90 + RSP
Original vs. Noisy					
	0.0927	0.0992	0.1177	0.1422	0.1822
Original vs. Denoised					
BM3D	0.0781 ± 0.067	0.0829 ± 0.064	0.1016 ± 0.061	0.1283 ± 0.056	0.1699 ± 0.051
DNCNN	0.0034 ± 0.003	0.0051 ± 0.002	0.0121 ± 0.014	0.0113 ± 0.002	0.0197 ± 0.004
FCAIDE	0.0011 ± 0.001	0.0030 ± 0.001	0.0053 ± 0.001	0.0073 ± 0.002	0.0090 ± 0.002
ADNET	0.0027 ± 0.002	0.0044 ± 0.001	0.0080 ± 0.002	0.0104 ± 0.003	0.0141 ± 0.003
BRDNet	0.0011 ± 0.001	0.0034 ± 0.001	0.0060 ± 0.001	0.0079 ± 0.002	0.0108 ± 0.003
GFF	0.0104 ± 0.009	0.0113 ± 0.004	0.0790 ± 0.230	0.0139 ± 0.008	0.0143 ± 0.004
RDUNET	0.0014 ± 0.000	0.0030 ± 0.001	0.0050 ± 0.001	0.0066 ± 0.002	0.0166 ± 0.006
TransUNET	0.0012 ± 0.001	0.0028 ± 0.001	0.0057 ± 0.001	0.0065 ± 0.002	0.0091 ± 0.002
HREDN	**0.0009 ± 0.000**	**0.0026 ± 0.001**	**0.0044 ± 0.001**	**0.0063 ± 0.002**	**0.0083 ± 0.002**

**Table 7:** Average PSNR gained before and after denoising CKPLUS images.

Methods	G:10 + RSP	G:30 + RSP	G:50 + RSP	G:70 + RSP	G:90 + RSP
Original vs. Noisy					
	11.7807	11.0194	9.8346	8.7665	7.5486
Original vs. Denoised					
BM3D	13.4348 ± 5.43	12.7514 ± 4.88	10.7952 ± 2.88	9.3025 ± 1.82	7.8825 ± 1.25
DNCNN	25.7057 ± 2.96	23.1300 ± 1.47	20.1699 ± 2.40	19.5756 ± 0.89	17.1669 ± 0.97
FCAIDE	29.8132 ± 1.70	25.4079 ± 1.13	22.9416 ± 1.16	21.4770 ± 1.08	20.6398 ± 1.20
ADNET	26.6098 ± 2.86	23.7323 ± 1.04	21.1195 ± 1.11	19.9613 ± 1.13	18.6523 ± 1.09
BRDNet	30.0759 ± 1.77	24.8131 ± 1.15	22.3180 ± 1.01	21.1683 ± 1.11	19.8075 ± 1.12
GFF	20.5565 ± 2.32	19.7845 ± 1.65	17.1302 ± 5.55	18.8690 ± 1.50	18.6391 ± 1.34
RDUNET	28.5416 ± 1.03	25.3700 ± 1.31	23.1461 ± 1.14	21.9464 ± 1.12	18.0431 ± 1.38
TransUNET	29.6529 ± 1.68	25.6144 ± 1.16	22.5530 ± 1.08	21.9911 ± 1.14	20.5479 ± 1.10
HREDN	**30.8059 ± 1.57**	**25.9801 ± 1.19**	**23.7090 ± 1.16**	**22.1735 ± 1.17**	**20.9619 ± 0.04**

**Table 8:** Average SSIM gained before and after denoising CKPLUS images.

Methods	G:10 + RSP	G:30 + RSP	G:50 + RSP	G:70 + RSP	G:90 + RSP
Original vs. Noisy					
	0.3960	0.3429	0.2750	0.2225	0.1734
Original vs. Denoised					
BM3D	0.4626 ± 0.21	0.4194 ± 0.19	0.3038 ± 0.11	0.2268 ± 0.08	0.1706 ± 0.06
DNCNN	0.8979 ± 0.05	0.8367 ± 0.05	0.7740 ± 0.05	0.7253 ± 0.05	0.5403 ± 0.05
FCAIDE	0.9707 ± 0.01	0.9201 ± 0.02	0.8702 ± 0.03	0.8258 ± 0.04	0.7870 ± 0.04
ADNET	0.9260 ± 0.03	0.8651 ± 0.03	0.7932 ± 0.06	0.7417 ± 0.04	0.6602 ± 0.04
BRDNet	0.9707 ± 0.01	0.9048 ± 0.02	0.8401 ± 0.03	0.8013 ± 0.04	0.7392 ± 0.04
GFF	0.7716 ± 0.07	0.7603 ± 0.04	0.6769 ± 0.16	0.7098 ± 0.05	0.7019 ± 0.05
RDUNET	0.9534 ± 0.01	0.9246 ± 0.02	0.8784 ± 0.03	0.8468 ± 0.03	0.6893 ± 0.05
TransUNET	0.9610 ± 0.01	0.9047 ± 0.03	0.8087 ± 0.05	0.8116 ± 0.05	0.7482 ± 0.05
HREDN	**0.9752 ± 0.01**	**0.9310 ± 0.02**	**0.8910 ± 0.02**	**0.8502 ± 0.03**	**0.8136 ± 0.04**

**Table 9:** Average IEF gained before and after denoising CKPLUS images.

Methods	G:10 + RSP	G:30 + RSP	G:50 + RSP	G:70 + RSP	G:90 + RSP
Original vs. Denoised					
BM3D	1.5722 ± 0.70	1.6895 ± 1.12	1.2670 ± 0.24	1.1334 ± 0.07	1.0803 ± 0.03
DNCNN	26.3715 ± 7.83	17.5795 ± 6.45	13.1186 ± 6.13	12.4262 ± 3.15	9.2680 ± 1.42
FCAIDE	74.0230 ± 34.27	31.1236 ± 14.53	21.6118 ± 7.07	19.2882 ± 5.00	20.8350 ± 4.53
ADNET	32.5192 ± 10.02	21.3975 ± 10.23	14.7096 ± 6.10	13.8772 ± 4.18	13.1702 ± 2.75
BRDNet	77.9218 ± 35.03	26.9789 ± 12.25	18.8929 ± 6.69	17.9399 ± 4.58	17.1437 ± 3.39
GFF	9.2810 ± 5.02	8.6178 ± 4.14	7.6207 ± 4.22	10.7420 ± 3.26	13.2688 ± 3.44
RDUNET	58.6180 ± 32.03	30.2517 ± 12.97	22.6860 ± 7.58	21.4818 ± 5.51	11.5312 ± 2.72
TransUNET	71.4136 ± 33.13	32.6235 ± 15.19	19.8365 ± 6.78	21.7372 ± 5.72	20.4034 ± 4.44
HREDN	**94.2450 ± 45.88**	**35.4397 ± 16.27**	**25.9209 ± 8.92**	**22.7043 ± 6.09**	**22.4347 ± 4.80**

**Table 10:** Average MSE gained before and after denoising Curated COVID CT dataset.

Methods	G:10 + RSP	G:30 + RSP	G:50 + RSP	G:70 + RSP	G:90 + RSP
Original vs. Noisy					
	0.0344	0.0426	0.0618	0.0886	0.1258
Original vs. Denoised					
BM3D	0.0240 ± 0.038	0.0266 ± 0.038	0.0402 ± 0.040	0.0695 ± 0.038	0.1103 ± 0.038
DNCNN	0.0119 ± 0.036	0.0031 ± 0.002	0.0140 ± 0.051	0.1205 ± 0.696	0.0113 ± 0.006
FCAIDE	0.0007 ± 0.001	0.0014 ± 0.001	0.0021 ± 0.001	0.0033 ± 0.002	0.0037 ± 0.002
ADNET	0.0020 ± 0.002	0.0045 ± 0.002	0.0070 ± 0.009	0.0064 ± 0.003	0.0098 ± 0.004
BRDNet	0.0008 ± 0.001	0.0016 ± 0.001	0.0033 ± 0.002	0.0039 ± 0.002	0.0037 ± 0.002
GFF	0.0104 ± 0.113	0.0220 ± 0.053	0.0066 ± 0.009	0.0079 ± 0.015	0.0105 ± 0.012
RDUNET	0.0012 ± 0.001	0.0062 ± 0.001	0.0022 ± 0.001	0.0028 ± 0.002	0.0046 ± 0.002
SwinUNET	0.0010 ± 0.001	0.0017 ± 0.001	0.0027 ± 0.002	0.0036 ± 0.002	0.0041 ± 0.003
HREDN	**0.0006 ± 0.001**	**0.0012 ± 0.001**	**0.0020 ± 0.001**	**0.0024 ± 0.001**	**0.0031 ± 0.002**

**Table 11:** Average PSNR gained before and after denoising Curated COVID CT dataset.

Methods	G:10 + RSP	G:30 + RSP	G:50 + RSP	G:70 + RSP	G:90 + RSP
Original vs. Noisy					
	16.5459	14.6544	12.5505	10.7434	9.1307
Original vs. Denoised					
BM3D	19.729 ± 5.60	18.690 ± 5.17	15.141 ± 2.88	11.958 ± 1.62	9.7560 ± 1.16
DNCNN	24.2760 ± 5.23	25.4330 ± 1.58	22.1402 ± 3.44	20.2527 ± 5.42	19.8496 ± 1.67
FCAIDE	32.6582 ± 2.36	29.0439 ± 1.83	27.2311 ± 1.88	25.2667 ± 1.84	24.7140 ± 1.69
ADNET	27.6839 ± 2.29	23.7655 ± 1.58	23.0164 ± 2.83	22.2420 ± 1.51	20.3353 ± 1.34
BRDNet	31.7221 ± 2.38	28.4166 ± 1.92	25.1626 ± 1.58	24.5054 ± 1.78	24.6908 ± 1.69
GFF	25.1290 ± 3.41	22.2717 ± 5.41	23.6673 ± 3.25	23.0447 ± 3.22	21.1637 ± 2.93
RDUNET	29.4405 ± 1.39	22.1498 ± 0.68	27.1482 ± 1.84	25.9761 ± 1.84	23.7356 ± 1.62
SwinUNET	30.7141 ± 2.25	28.3595 ± 1.95	26.2044 ± 1.94	25.0414 ± 1.98	24.3525 ± 1.91
HREDN	**33.0712 ± 2.44**	**29.8158 ± 2.04**	**27.5033 ± 1.83**	**26.6562 ± 1.83**	**25.5341 ± 1.73**

**Table 12:** Average SSIM gained before and after denoising Curated COVID CT dataset.

Methods	G:10 + RSP	G:30 + RSP	G:50 + RSP	G:70 + RSP	G:90 + RSP
Original vs. Noisy					
	0.4942	0.3082	0.2031	0.1423	0.1019
Original vs. Denoised					
BM3D	0.5756 ± 0.17	0.5143 ± 0.15	0.2883 ± 0.06	0.1431 ± 0.04	0.0940 ± 0.03
DNCNN	0.7776 ± 0.09	0.8144 ± 0.04	0.6974 ± 0.06	0.5203 ± 0.08	0.5188 ± 0.04
FCAIDE	0.9543 ± 0.02	0.9103 ± 0.04	0.8795 ± 0.04	0.8348 ± 0.05	0.8178 ± 0.05
ADNET	0.8856 ± 0.03	0.6902 ± 0.07	0.7736 ± 0.12	0.5641 ± 0.08	0.5052 ± 0.06
BRDNet	0.9433 ± 0.03	0.8933 ± 0.04	0.8130 ± 0.04	0.8018 ± 0.05	0.8186 ± 0.05
GFF	0.8232 ± 0.08	0.6994 ± 0.19	0.8033 ± 0.09	0.7797 ± 0.07	0.7112 ± 0.11
RDUNET	0.8750 ± 0.02	0.6031 ± 0.05	0.8740 ± 0.04	0.8477 ± 0.05	0.7920 ± 0.05
SwinUNET	0.8958 ± 0.04	0.8326 ± 0.05	0.7731 ± 0.06	0.7337 ± 0.07	0.7115 ± 0.07
HREDN	**0.9592 ± 0.02**	**0.9176 ± 0.03**	**0.8855 ± 0.04**	**0.8689 ± 0.04**	**0.8387 ± 0.05**

**Table 13:** Average IEF gained before and after denoising Curated COVID CT dataset.

Methods	G:10 + RSP	G:30 + RSP	G:50 + RSP	G:70 + RSP	G:90 + RSP
Original vs. Denoised					
BM3D	2.2878 ± 1.07	3.2563 ± 2.88	1.8942 ± 0.67	1.3306 ± 0.16	1.1563 ± 0.06
DNCNN	6.6344 ± 2.77	12.9500 ± 5.82	10.2342 ± 3.03	11.5061 ± 4.18	12.1719 ± 3.12
FCAIDE	51.4136 ± 38.61	30.6735 ± 16.86	31.0313 ± 11.23	29.6048 ± 8.98	37.9663 ± 11.76
ADNET	14.5153 ± 6.13	8.7725 ± 3.72	11.7021 ± 3.35	14.3738 ± 2.70	13.5151 ± 3.01
BRDNet	39.8532 ± 26.14	26.2605 ± 13.43	18.9899 ± 5.89	24.6910 ± 6.73	37.6310 ± 11.10
GFF	9.9782 ± 6.98	7.2333 ± 3.28	14.1050 ± 4.56	19.3547 ± 8.08	17.6712 ± 7.24
RDUNET	24.9305 ± 17.65	6.9188 ± 5.29	30.5303 ± 11.70	35.2578 ± 12.59	30.2938 ± 9.68
SwinUNET	32.0464 ± 22.72	26.4815 ± 15.78	24.8669 ± 10.91	28.4843 ± 10.36	35.3092 ± 12.43
HREDN	**56.4491 ± 42.18**	**36.9480 ± 21.78**	**33.4629 ± 14.28**	**41.1711 ± 14.35**	**46.1834 ± 15.93**

**Table 14:** Average MSE gained before and after denoising NWPU-RESISC45 dataset.

Methods	G:10 + RSP	G:30 + RSP	G:50 + RSP	G:70 + RSP	G:90 + RSP
Original vs. Noisy					
	0.0463	0.0549	0.0732	0.1001	0.1379
Original vs. Denoised					
BM3D	0.0310 ± 0.048	0.0352 ± 0.049	0.0517 ± 0.049	0.0825 ± 0.046	0.1232 ± 0.046
DNCNN	0.0025 ± 0.002	0.0049 ± 0.013	0.0143 ± 0.093	0.0619 ± 0.332	0.0089 ± 0.005
FCAIDE	0.0009 ± 0.001	0.0017 ± 0.002	0.0026 ± 0.002	0.0034 ± 0.003	0.0044 ± 0.004
ADNET	0.0024 ± 0.001	0.0037 ± 0.004	0.0034 ± 0.003	0.0060 ± 0.004	0.0064 ± 0.005
BRDNet	0.0015 ± 0.001	0.0020 ± 0.002	0.0286 ± 0.024	0.0038 ± 0.003	0.0059 ± 0.005
GFF	0.0031 ± 0.004	0.0623 ± 0.158	0.1029 ± 0.522	2.5468 ± 16.767	0.0182 ± 0.031
RDUNET	**0.0007 ± 0.001**	0.0017 ± 0.002	0.0026 ± 0.003	0.0315 ± 0.006	0.0493 ± 0.009
SwinUNET	0.0008 ± 0.001	0.0018 ± 0.002	0.0028 ± 0.003	0.0035 ± 0.004	0.0043 ± 0.005
HREDN	**0.0007 ± 0.001**	**0.0016 ± 0.001**	**0.0025 ± 0.002**	**0.0033 ± 0.003**	**0.0039 ± 0.004**

**Table 15:** Average PSNR gained before and after denoising NWPU-RESISC45 dataset.

Methods	G:10 + RSP	G:30 + RSP	G:50 + RSP	G:70 + RSP	G:90 + RSP
Original vs. Noisy					
	15.3335	13.7215	11.9126	10.2763	8.7689
Original vs. Denoised					
BM3D	19.3923 ± 6.50	18.2901 ± 6.22	14.2067 ± 3.23	11.2872 ± 1.82	9.3137 ± 1.29
DNCNN	26.9817 ± 2.87	25.0395 ± 3.34	23.6504 ± 3.79	21.3229 ± 5.54	21.0595 ± 1.96
FCAIDE	31.4057 ± 2.60	28.8960 ± 3.14	26.9885 ± 3.05	26.0518 ± 3.18	24.8383 ± 3.14
ADNET	26.8334 ± 2.09	25.1769 ± 2.48	25.7991 ± 2.80	22.8546 ± 2.11	22.6764 ± 2.26
BRDNet	28.9446 ± 2.31	28.2385 ± 3.00	16.2222 ± 2.30	25.4023 ± 2.92	23.2963 ± 2.65
GFF	27.4096 ± 4.27	22.0400 ± 8.49	20.2756 ± 6.74	21.8686 ± 10.31	20.6686 ± 4.54
RDUNET	32.7698 ± 3.25	28.9744 ± 3.25	27.2181 ± 3.30	15.0838 ± 0.79	13.1487 ± 0.81
SwinUNET	32.4972 ± 3.14	28.5223 ± 2.94	26.7357 ± 3.08	26.0650 ± 3.42	25.3101 ± 3.67
HREDN	**32.7986 ± 3.16**	**29.2580 ± 3.26**	**27.3235 ± 3.30**	**26.1450 ± 3.28**	**25.5101 ± 3.33**

**Table 16:** Average SSIM gained before and after denoising NWPU-RESISC45 dataset.

Methods	G:10 + RSP	G:30 + RSP	G:50 + RSP	G:70 + RSP	G:90 + RSP
Original vs. Noisy					
	0.2836	0.1915	0.1265	0.0882	0.0634
Original vs. Denoised					
BM3D	0.4762 ± 0.24	0.3739 ± 0.22	0.1334 ± 0.09	0.0705 ± 0.06	0.0475 ± 0.05
DNCNN	0.7976 ± 0.07	0.7575 ± 0.08	0.6770 ± 0.09	0.6245 ± 0.10	0.5376 ± 0.07
FCAIDE	0.9126 ± 0.03	0.8566 ± 0.07	0.8087 ± 0.07	0.7798 ± 0.08	0.7565 ± 0.10
ADNET	0.8510 ± 0.05	0.7604 ± 0.07	0.7707 ± 0.08	0.6472 ± 0.06	0.5696 ± 0.07
BRDNet	0.8530 ± 0.04	0.8455 ± 0.06	0.2623 ± 0.08	0.7576 ± 0.08	0.6801 ± 0.09
GFF	0.8238 ± 0.09	0.6437 ± 0.27	0.6921 ± 0.18	0.6995 ± 0.20	0.6936 ± 0.15
RDUNET	0.9321 ± 0.04	0.8547 ± 0.07	0.8114 ± 0.08	0.2280 ± 0.08	0.1620 ± 0.07
SwinUNET	0.8557 ± 0.07	0.7271 ± 0.11	0.6499 ± 0.11	0.6040 ± 0.11	0.5704 ± 0.13
HREDN	**0.9328 ± 0.04**	**0.8652 ± 0.06**	**0.8158 ± 0.08**	**0.7889 ± 0.09**	**0.7631 ± 0.10**

**Table 17:** Average IEF gained before and after denoising NWPU-RESISC45 dataset.

Methods	G:10 + RSP	G:30 + RSP	G:50 + RSP	G:70 + RSP	G:90 + RSP
Original vs. Denoised					
BM3D	3.0925 ± 2.28	4.2752 ± 5.17	1.7778 ± 0.57	1.2686 ± 0.13	1.1348 ± 0.05
DNCNN	16.1654 ± 6.99	14.5267 ± 4.57	17.6271 ± 7.52	17.0917 ± 7.45	18.1111 ± 6.86
FCAIDE	55.2539 ± 0.03	43.7731 ± 42.69	39.1788 ± 29.14	45.7072 ± 30.07	47.6427 ± 26.99
ADNET	16.4873 ± 9.09	16.8464 ± 11.29	29.5449 ± 21.09	20.0266 ± 9.96	26.5407 ± 9.55
BRDNet	27.7074 ± 16.54	34.1582 ± 22.46	2.7167 ± 0.32	37.4862 ± 19.71	31.3183 ± 12.30
GFF	23.9413 ± 21.58	22.7844 ± 27.40	11.9775 ± 8.65	30.5923 ± 23.35	20.0421 ± 11.57
RDUNET	83.3028 ± 93.30	45.8627 ± 49.66	42.8922 ± 35.81	3.2826 ± 1.61	2.8931 ± 1.15
SwinUNET	76.9846 ± 85.22	41.7133 ± 52.54	38.3050 ± 33.11	47.4897 ± 34.78	58.0151 ± 43.56
HREDN	**84.3325 ± 96.60**	**49.3666 ± 55.75**	**43.3928 ± 33.26**	**47.8326 ± 34.86**	**58.0657 ± 37.55**

**Table 18:** Statistical evaluation of HREDN performance across domains.

Metric	ANOVA *p*-Value	Levene’s *p*-Value
MSE	0.0963	0.0756
PSNR	0.1585	0.8916
SSIM	0.3641	0.4112
IEF	0.3520	0.7268

**Table 19:** Impact of modules on network performance for denoising Fer2013 images with noise (G:30 + RSP).

Methods	Average MSE	Average PSNR	Average SSIM	Average IEF
ED	0.0028 ± 0.00	25.7280 ± 1.52	0.8919 ± 0.03	33.3376 ± 20.67
ED + Attention	0.0028 ± 0.00	25.7320 ± 1.55	0.8943 ± 0.03	33.2194 ± 20.42
ED + Attention + MSFEB	**0.0027 ± 0.00**	**25.8967 ± 1.52**	**0.8951 ± 0.03**	**34.6337 ± 21.51**

**Table 20:** Impact of modules on network performance for denoising Fer2013 images.

Methods	Training Time (Minutes)	Number of Parameters (Millions)	Inference Time (Seconds)
ED	39	**38.38**	20
ED + Attention	**27**	41.53	**19**
ED + Attention + MSFEB	63	60.68	27

## Data Availability

Data that are available in publicly accessible repositories do not issue DOIs. The used data can be found here: the Facial Expression Recognition 2013 Dataset (FER2013) is available at https://www.kaggle.com/datasets/msambare/fer2013 (accessed on 31 December 2019), the Cohn-Kanade Plus 48 (CKPLUS-48) dataset is available at https://sites.pitt.edu/~emotion/ck-spread.htm (accessed on 15 April 2020), the Curated COVID CT dataset is available at https://www.kaggle.com/datasets/maedemaftouni/large-covid19-ct-slice-dataset (accessed on 22 May 2024), and the NWPU-RESISC25 dataset is available at https://www.kaggle.com/datasets/aaryaankurparikh/nwpu-resisc25 (accessed on 28 January 2025).

## Abbreviations

The following abbreviations are used in this manuscript:

HREDN	Highly Robust Encoder–Decoder Network
CNN	Convolutional Neural Network
CT	Computed Tomography
MSE	Mean Squared Error
PSNR	Peak Signal-to-Noise Ratio
SSIM	Structural Similarity Index Measure
IEF	Image Enhancement Framework
G	Gaussian
RSP	Random Salt-and-Pepper
MSFEB	Multi-Scale Feature Enhancement Block
FER2013	Facial Expression Recognition 2013
CKPLUS-48	Cohn-Kanade Plus Dataset, 48 × 48 Resolution
BM3D	Block-Matching and 3D Filtering
DNCNN	Denoising Convolutional Neural Network
FCAIDE	Fully Convolutional Pixel Adaptive Image Denoise
ADNET	Attention-Guided CNN for Image Denoising
BRDNET	Batch-Renormalization Denoising Network
RDUNET	Residual Dense Neural Network
ED	Encoder–Decoder
ReLU	Rectified Linear Unit

## References

[1] Wang M., Zheng S., Li X., Qin X. A New Image Denoising Method Based on Gaussian Filter. Proceedings of the 2014 International Conference on Information Science, Electronics and Electrical Engineering.

[2] Li X., Ji J., Li J., He S., Zhou Q. Research on Image Denoising Based on Median Filter. Proceedings of the IMCEC 2021—IEEE 4th Advanced Information Management, Communicates, Electronic and Automation Control Conference.

[3] Bhonsle D., Chandra V., Sinha G.R. (2012). Medical Image Denoising Using Bilateral Filter. Int. J. Image Graph. Signal Process..

[4] Dabov K., Foi A., Katkovnik V., Egiazarian K. Image Denoising with Block-Matching and 3D Filtering. Proceedings of the Image Processing: Algorithms and Systems, Neural Networks, and Machine Learning.

[5] Tian C., Fei L., Zheng W., Xu Y., Zuo W., Lin C.W. (2020). Deep Learning on Image Denoising: An Overview. Neural Netw..

[6] Ilesanmi A.E., Ilesanmi T.O. (2021). Methods for Image Denoising Using Convolutional Neural Network: A Review. Complex. Intell. Syst..

[7] Ghose S., Singh N., Singh P. Image Denoising Using Deep Learning: Convolutional Neural Network. Proceedings of the Confluence 2020—10th International Conference on Cloud Computing, Data Science and Engineering.

[8] Ronneberger O., Fischer P., Brox T. U-Net: Convolutional Networks for Biomedical Image Segmentation. Proceedings of the Lecture Notes in Computer Science (Including Subseries Lecture Notes in Artificial Intelligence and Lecture Notes in Bioinformatics).

[9] Oktay O., Schlemper J., Le Folgoc L., Lee M., Heinrich M., Misawa K., Mori K., Mcdonagh S., Hammerla N.Y., Kainz B. (2018). Attention U-Net: Learning Where to Look for the Pancreas. arXiv.

[10] Zhang K., Zuo W., Chen Y., Meng D., Zhang L. (2017). Beyond a Gaussian Denoiser: Residual Learning of Deep CNN for Image Denoising. IEEE Trans. Image Process..

[11] Cha S., Moon T. Fully Convolutional Pixel Adaptive Image Denoiser. Proceedings of the 2019 IEEE/CVF International Conference on Computer Vision (ICCV).

[12] Tian C., Xu Y., Li Z., Zuo W., Fei L., Liu H. (2020). Attention-Guided CNN for Image Denoising. Neural Netw..

[13] Tian C., Xu Y., Zuo W. (2020). Image Denoising Using Deep CNN with Batch Renormalization. Neural Netw..

[14] Li X., Zhao H., Han L., Tong Y., Yang K. (2019). GFF: Gated Fully Fusion for Semantic Segmentation. Proc. AAAI.

[15] Gurrola-Ramos J., Dalmau O., Alarcón T.E. (2021). A Residual Dense U-Net Neural Network for Image Denoising. IEEE Access.

[16] Zhang H., Lian Q., Zhao J., Wang Y., Yang Y., Feng S. (2022). RatUNet: Residual U-Net Based on Attention Mechanism for Image Denoising. PeerJ Comput. Sci..

[17] Mafi M., Izquierdo W., Martin H., Cabrerizo M., Adjouadi M. (2020). Deep Convolutional Neural Network for Mixed Random Impulse and Gaussian Noise Reduction in Digital Images. IET Image Process.

[18] Khmag A. (2023). Additive Gaussian Noise Removal Based on Generative Adversarial Network Model and Semi-Soft Thresholding Approach. Multimed. Tools Appl..

[19] Dantas C.F., Da Costa M.N., Da Rocha Lopes R. (2017). Learning Dictionaries as a Sum of Kronecker Products. IEEE Signal Process Lett..

[20] Khmag A., Kamarudin N. Natural Image Deblurring Using Recursive Deep Convolutional Neural Network (R-DbCNN) and Second-Generation Wavelets. Proceedings of the 2019 IEEE International Conference on Signal and Image Processing Applications, ICSIPA.

[21] Hasan M., El-Sakka M.R. (2018). Improved BM3D Image Denoising Using SSIM-Optimized Wiener Filter. EURASIP J. Image Video Process.

[22] Min C., Wen G., Li B., Fan F. (2018). Blind Deblurring via a Novel Recursive Deep CNN Improved by Wavelet Transform. IEEE Access.

[23] Xu L., Ren J.S.J., Liu C., Jia J. Deep Convolutional Neural Network for Image Deconvolution. Proceedings of the 28th International Conference on Neural Information Processing Systems (NIPS).

[24] Cheng S., Zhuang Y., Kahouadji L., Liu C., Chen J., Matar O.K., Arcucci R. (2024). Multi-Domain Encoder–Decoder Neural Networks for Latent Data Assimilation in Dynamical Systems. Comput. Methods Appl. Mech. Eng..

[25] Zhou H., Cheng S., Arcucci R. (2024). Multi-Fidelity Physics Constrained Neural Networks for Dynamical Systems. Comput. Methods Appl. Mech. Eng..

[26] Chen J., Lu Y., Yu Q., Luo X., Adeli E., Wang Y., Lu L., Yuille A.L., Zhou Y. (2021). TransUNet: Transformers Make Strong Encoders for Medical Image Segmentation. arXiv.

[27] He K., Zhang X., Ren S., Sun J. Deep Residual Learning for Image Recognition. Proceedings of the 2016 IEEE Conference on Computer Vision and Pattern Recognition (CVPR).

[28] Korhonen J., You J. Peak Signal-to-Noise Ratio Revisited: Is Simple Beautiful?. Proceedings of the 2012 4th International Workshop on Quality of Multimedia Experience.

[29] Wang Z., Bovik A.C., Sheikh H.R., Simoncelli E.P. (2004). Image Quality Assessment: From Error Measurement to Structural Similarity. IEEE Trans. Image Process..

[30] Nair V., Hinton G.E. Rectified Linear Units Improve Restricted Boltzmann Machines. Proceedings of the 27th International Conference on Machine Learning (ICML).

[31] Goodfellow I.J., Erhan D., Luc Carrier P., Courville A., Mirza M., Hamner B., Cukierski W., Tang Y., Thaler D., Lee D.H. (2015). Challenges in Representation Learning: A Report on Three Machine Learning Contests. Neural Netw..

[32] Kanade T., Cohn J.F., Tian Y. Comprehensive Database for Facial Expression Analysis. Proceedings of the 4th IEEE International Conference on Automatic Face and Gesture Recognition.

[33] Maftouni M., Law A.C.C., Shen B., Kong Grado Z., Zhou Y., Yazdi N.A. A Robust Ensemble-Deep Learning Model for COVID-19 Diagnosis Based on an Integrated CT Scan Images Database. Proceedings of the IISE Annual Conference and Expo 2021.

[34] Cheng G., Han J., Lu X. (2017). Remote Sensing Image Scene Classification: Benchmark and State of the Art. Proc. IEEE.

[35] Kingma D.P., Ba J.L. Adam: A Method for Stochastic Optimization. Proceedings of the 3rd International Conference on Learning Representations (ICLR).

[36] Cao H., Wang Y., Chen J., Jiang D., Zhang X., Tian Q., Wang M. Swin-Unet: Unet-Like Pure Transformer for Medical Image Segmentation. Proceedings of the Lecture Notes in Computer Science (Including Subseries Lecture Notes in Artificial Intelligence and Lecture Notes in Bioinformatics).

